# Oscillatory differentiation dynamics fundamentally restricts the resolution of pseudotime reconstruction algorithms

**DOI:** 10.1098/rsif.2023.0537

**Published:** 2024-03-20

**Authors:** Huy K. Vo, Jonathan H. P. Dawes, Robert N. Kelsh

**Affiliations:** ^1^ Department of Mathematical Sciences, University of Bath, BA2 7AY Bath, UK; ^2^ Department of Life Sciences, University of Bath, BA2 7AY Bath, UK

**Keywords:** development, genetic regulation, multi-potency, single-cell RNA sequencing

## Abstract

The challenge to understand differentiation and cell lineages in development has resulted in many bioinformatics software tools, notably those working with gene expression data obtained via single-cell RNA sequencing obtained at snapshots in time. Reconstruction methods for trajectories often proceed by dimension reduction, data clustering and then computation of a tree graph in which edges indicate closely related clusters. Cell lineages can then be deduced by following paths through the tree. In the case of multi-potent cells undergoing differentiation, this trajectory reconstruction involves the reconstruction of multiple distinct lineages corresponding to commitment to each of a set of distinct fates. Recent work suggests that there may be cases in which the cell differentiation process involves trajectories that explore, in a dynamic and oscillatory fashion, propensity to differentiate into a number of possible cell fates before commitment finally occurs. Here, we show theoretically that the presence of such oscillations provides intrinsic constraints on the quality and resolution of the trajectory reconstruction process, even for idealized noise-free data. These constraints point to inherent common limitations of current methodologies and serve both to provide additional challenge in the development of software tools and also may help to understand features observed in recent experiments.

## 1. Introduction

The detailed mechanisms by which cells differentiate to form tissues and organs is a central focus of modern systems biology, requiring the integration of data of many different kinds across time, space and function. The resulting complexity of molecular biology has invited increasing interaction with physical and mathematical ideas which continue both to provide organizing principles and to generate hypotheses that can then be tested experimentally [1].

The continuing development of single-cell RNA sequencing (scRNA-seq) has in recent years provided a wealth of detailed data on messenger RNA (mRNA) expression levels that allow us to infer the levels of gene activity relevant to cellular processes [2]. The scRNA-seq technique is particularly valuable in the context of our understanding of how stem cells behave and the elucidation of the genetic regulatory networks that underpin their function and ability to differentiate. While the typical throughput of scRNA-seq analyses has increased by several orders of magnitude, datasets are still subject to various sources of bias, noise and missing data. These issues remain challenges that stimulate both future development of laboratory equipment and technique, but also statistical methods for understanding and handling scRNA-seq datasets.

A brief outline of the typical data processing protocol that starts with scRNA-seq data and aims to deduce cell dynamics, sufficient to set the scene here and highlight the complexity of the process while ignoring many experimental and biological details, is as follows [3,4]. First one acquires, at one or more moments in time, a set of *n* cells whose diversity is thought to follow the different developmental stages that arise along one or more trajectories of cellular development. Then levels of gene expression are measured via the concentration of relevant mRNAs. Standard techniques for scRNA-seq are not focused on specific gene classes and their sensitivity is limited by the depth of the sequencing. Among this (large) collection of expressed genes, the search for transcription factors (TFs), i.e. the key classes of protein that play central roles in regulating the differentiation process, is challenging since the genes that code for them are likely to be expressed at very low levels compared with other classes of gene such as those involved in the cell cycle.

To elaborate on this point a little further, in fact TFs (in combination with cell surface receptors) play key roles in determining the formation of different cell types in many situations including in development. But despite their importance, levels of gene expression for TFs are notoriously difficult to measure in single-cell transcriptomics due to the low concentration of their mRNA. Hence in practice in many cases experimentalists observe instead the activity of ‘marker genes’ which are more or less specific to particular cell types and whose transcriptional activity is expected to be highly correlated with that of the genes which code for the TFs themselves. The direct measurement of the transcriptional activity of TFs is a topic of considerable current interest. Further, TFs themselves may be activated by other processes, such as phosphorylation [5], rather than just interacting with each other.

Suppose now that we have obtained the expression levels for a collection of *p* marker genes. The *p* × *n* matrix of expression data is interpreted as a collection of *n* points embedded in the (high-dimensional) state space $R^{p}$. The dimension of this state space is reduced through methods such as principal component analysis or t-stochastic neighbour embedding. After the data points are effectively projected into a lower-dimensional space, we are able to focus on the specific marker genes which are supposed to indicate transcriptional activity of TFs that drive the developmental process of interest. Through clustering we implicitly assume that points lying close to each other correspond to cells that are evolving at similar points on their dynamical developmental journey. Treating the clusters themselves as nodes in an undirected graph, a minimum spanning tree can be generated in which proximal clusters are joined by edges, while avoiding the formation of closed cycles in the graph in order to ensure that a well-defined sense of ‘time evolution’ can be inferred from the data. Paths through the graph are then deemed to correspond to distinct developmental lineages along which at least some fraction of the original set of cells are assumed to have evolved.

Often it is straightforward to identify an origin leaf-node for developmental paths, and then, due to the tree structure, there is a unique path connecting each other leaf to the origin. Each path through the graph corresponds to an ordered subset of the clusters—these are referred to as cell ‘lineages’. The points in that set of clusters can be used to construct a principal curve through that subset of the data, together with a ‘time-like’ arclength assignment that is generally known as a ‘pseudotime’. Pseudotimes are therefore a proxy for true developmental time, remembering always that the scRNA-seq data is obtained at a single point in real time due to the experimental protocol, and that cells start their developmental trajectory earlier or later in real time and so represent different time points in the snapshot data. Despite its successes and insights, it is clear that the process of correlating pseudotime with developmental stage is fraught with implicit and problematic issues. These issues range from the fundamental to subtle questions of how best to design and tune algorithms for processing single-cell data. An example of a fundamental issue is the question raised in [6], as to whether cell fate transitions are actually discontinuous stochastic events rather than smooth and continuous as one would often like to assume. Tsuchiya *et al*. [7] discuss an alternative viewpoint in which a cell fate transition is described as a ‘critical point’ for the cell as a complex system, in the sense of self-organized criticality. In our discussion here, we implicitly assume that cell fate transitions are at least smooth enough that the pseudotime-based reconstruction process makes sense.

On the data processing side, there is now at least two decades of development of temporal reconstruction methods in general, dating back at least as far as 2003 [8], and there are now many methods, and many variations on these methods, available (see, for example [3,9,10]). Indeed, the authors of [10] state that over 70 trajectory inference tools have been developed, out of which they review and compare a subset of 45. While we do not attempt to provide a comprehensive review here, it is worth noting that, given the issues around the notion of pseudotime, one important strand of work has been to develop probabilistic modelling approaches to test the robustness of estimates of pseudotime. This robustness can be explored by, for example, re-estimating pseudotimes by sampling multiple random subsets of the full dataset. When data is available at multiple points in real time, the resulting ‘repeated cross-sectional data’ can be used not only to estimate the uncertainty in pseudotime estimates, but also to connect the idea of pseudotime much more closely to real developmental time—pseudotime otherwise is not necessarily grounded in any direct relation to laboratory time. Data collected at multiple time points is highly likely to help reveal the asynchronous nature of the underlying biology, and this in turn improves estimates of pseudotime. Statistical inference schemes based on Gaussian processes for uncertainty estimation in pseudotimes have been developed in [11] (implemented in the DeLorean package) and in the GrandPrix package developed in [12].

In this paper, we focus on the clustering and lineage construction steps in the protocol outlined above, for snapshot data obtained at a single point in time, and make use of the routines from the widely used package *slingshot* [13] implemented in the open-source statistical software R. Overall, *slingshot* is a collection of bioinformatics tools for the reconstruction of dynamical behaviour at a cellular level from scRNA-seq data. Here, our aim is to explore the behaviour of the mathematical methods that underpin this pseudotime reconstruction algorithm when it is confronted with data that describe cellular differentiation taking place in an oscillatory fashion rather than the monotonic separation of cell lineages that is usually implicitly expected to take place [10,13–15]. In order to test the algorithm, we generate synthetic data from a set of model differential equations that describe our novel cyclical fate restriction (CFR) model [16] since this contains a parameter that allows the level of oscillation in the dynamics to be directly varied without altering other aspects of the model. The data produced from the conceptual model is likely not to reflect in detail the statistical properties of real gene expression data; this is a key caveat in our approach that needs to be borne in mind throughout. We note that the topic of oscillatory gene expression arises in many contexts; of particular note is work on the Delta-Notch system [17,18] and the review article [19].

For further details of our specific model system, we refer the reader to recent papers [16,20–22] which summarize the biological background, recent experimental work and mathematical modelling. Here, our focus is purely on the potential for the oscillatory nature of developmental trajectories to destabilize pseudotime reconstruction methodologies, and not on the specific biological details of this model gene regulatory system. Our aim is rather to explore whether aspects of the kinds of algorithm built into *slingshot* and similar software can successfully reconstruct the known ‘ground truth’ set of lineages that our model represents.

We find overall that temporal oscillations in gene expression levels provide a significant barrier to successful lineage reconstruction, even if noise levels in the data are very low, and that typical clustering algorithms such as *k*-means work in ways that limit their ability to detect the presence of oscillatory dynamics. Therefore, it is probable that these software packages are not able to detect the presence of oscillations of the kind that we have hypothesized, and that this is a fundamental challenge to the current precision of scRNA-seq methods. We use the *k*-means clustering algorithm here since it is widely understood and we can control directly the number of clusters formed. In our concluding section, we comment further on the limitations of *k*-means and its relation to other algorithms used in the field, and we argue that our essential message is independent of the choice of clustering algorithm.

The remainder of this paper is organized as follows. In §2, we summarize the underlying conceptual model. Section 3 explains our procedure for the generation of the synthetic data and the clustering and lineage generation steps which are central to *slingshot* and many other lineage reconstruction tools. Our results are presented in §4 and reveal that the generation of anomalous short lineages is a more serious issue than the artificial generation of multiple longer lineages. This motivates the construction and analysis in §5 of a conceptual mathematical model that can be completely solved analytically. The model helps to explain the conditions under which these anomalous short lineages appear in the analysis of scRNA-seq data and why this is inherently the case if the underlying dynamics have a sufficiently strongly oscillatory character. Conclusions are presented in §6.

## 2. A conceptual model for cellular differentiation

In our conceptual model and the remainder of the paper, we will refer to TFs even though, in the light of the discussion above, these will in real life be represented by data for marker genes of the developmental process.

We propose a low-dimensional model in which expression levels of TFs interact dynamically and we use it to generate synthetic data, thus bypassing the initial data processing steps common to scRNA-seq analysis in which key relevant TFs are identified and a low-dimensional representation of the data is produced, for example via t-SNE [23] or UMAP [24]. The review article by [25] contains extended discussion of both of these methods and variations on them (for example [26]), highlighting the challenges in this initial step of producing a low-dimensional representation of initially high-dimensional data which we avoid through our use of low-dimensional synthetic data in order to focus only on the issue of oscillatory dynamical features.

We then systematically vary a parameter (denoted by *α* below) that describes the amount of ‘twisting’ that trajectories undergo before their eventual (monotonic) convergence to the neighbourhood of a new equilibrium point that signifies a differentiated cellular state. We show that the twisting presents challenges to the trajectory reconstruction algorithm in the sense that the usual *slingshot* procedure is prone to generate spurious additional ‘lineages’ that were not present in the synthetic model, the dynamics of which are completely understood, and which therefore represents the underlying ground truth. The generation of these spurious additional lineages can be prevented by clustering the data more coarsely, into a smaller number of larger clusters, and this inevitably reduces the level of detail then available in the results. These findings occur even in the noise-free case, and, as one might imagine, the problem of the existence of spurious lineages only becomes more challenging as the noise level in the synthetic data is increased.

**Figure 1.**
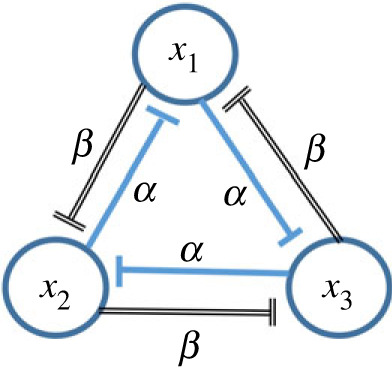
Schematic illustration of the GRN that forms the basis for our conceptual model for cyclical fate restriction (CFR), comprising three TFs that mutually inhibit each other. The parameters *α* (blue inhibitory arrows) and *β* (black inhibitory arrows) describe the intensity of the cross-repressive inhibitory influences in the two directed rings of inhibitory couplings.

Our conceptual model for cell differentiation describes the state of an initially multi-potent cell via the levels of three TFs, each of which regulates the production of the other two via a simple gene regulatory network (GRN) that we term the ‘cross-repressilator’ since it is similar to two copies of the ‘repressilator’ network introduced in [27]. Instead of a single cyclical set of three inhibitory links, the cross-repressilator contains two sets of cyclical inhibitory influences, allowing each TF to be inhibited by the presence of either of the others, with different strengths of inhibition allowed in each set. Each of these TFs is considered to be a master regulator of a specific cell-type. This GRN is illustrated schematically in figure 1.

Our particular conceptual model arises from previous work in which we attempt to reconcile two existing paradigms for fate restriction—the process through which an initially multi-potent stem cell eventually chooses one specific cell fate. These paradigms are known as ‘direct fate restriction’ (DFR) and ‘progressive fate restriction’ (PFR). The key difference between DFR and PFR is the question of the presence or absence of any intermediate cell types that are ‘partially restricted’ in their fates. If intermediates exist, then the PFR paradigm, where successive branches in possible cell fates should be observable, feels more correct. If, instead, there are no such intermediates, then the DFR model, in which cells make only one decision, and move towards one of a number of fates at the same developmental point, feels more correct. In the existing literature on these two routes, there appears to be an implicit assumption that the process of differentiation would take place in a ‘monotonic’ way, in that levels of the relevant TFs would either increase or decrease from the multi-potent state, until a new equilibrium was reached that would correspond to one of the several possible choices of fate.

The biological context and motivation for this modelling work is the neural crest in zebrafish which is responsible, at approximately 2–3 days post-fertilization, for the generation of the coloured cells that are characteristic of zebrafish: melanophores which contain black pigment, xanthophores which contain yellow pigment, and iridophores which exhibit structural colours and are silver in the embryo (and silver or blue in adults), as well as glial and neuronal cell types.

In dynamical terms, the CFR conceptual model is unique and distinctive in that the transition from multi-potent to fate-restricted states is mediated through an oscillatory state in which trajectories of the dynamical system, corresponding to developmental paths, make a series of visits to the neighbourhoods of each of the differentiated states before eventually fixing on one of these ‘sub-states’ to settle towards. Which ‘sub-state’ is preferred depends on the initial condition for the trajectory, fate determining environmental signals (here represented by a single external input *g*(*t*)), and, likely, the effect of noise. We now turn to a specific dynamical system that generates the inhibitory interactions shown in figure 1 and from which we will generate our synthetic data.

### 2.1. The stochastic differential equation model for cyclical fate restriction

A minimal model of ordinary differential equations (ODEs) describing the cyclical fate restriction hypothesis was proposed in [16] and investigated in detail in [21]. In the model, the state of each cell is given by the levels (*x*_1_(*t*), *x*_2_(*t*), *x*_3_(*t*)) of the three TFs which evolve according to the equations

2.1 $$\frac{dx_{1}}{dt}=b+\frac{g(t)}{\left(1+\alpha x_{2}^{h})(1+\beta x_{3}^{h}\right)}-dx_{1},$$

2.2 $$\frac{dx_{2}}{dt}=b+\frac{g(t)}{\left(1+\alpha x_{3}^{h})(1+\beta x_{1}^{h}\right)}-dx_{2}$$

2.3 $$\mathrm{and}\;\;\;\;\;\;\;\;\;\;\frac{dx_{3}}{dt}=b+\frac{g(t)}{\left(1+\alpha x_{1}^{h})(1+\beta x_{2}^{h}\right)}-dx_{3}.$$

On the right-hand side of each equation the first term *b* represents a constant (low) level of production of the TF, the second term represents the inhibitory influences of the other two TFs, motivated by the usual Hill function form with exponent *h*, hence a ‘cross-repressilator’, and the final term −*dx*_*i*_ represents a natural rate of degradation of the TF *x*_*i*_. The inhibitory term in the ODE for a given variable *x*_*i*_ is small whenever the level of either of the other TFs is large, which leads us to call this formulation an ‘OR gate’. In terms of the GRN shown in figure 1, this corresponds to each inhibitory influence acting independently. All the parameters are taken to be positive, and for simplicity, we enforce a cyclical symmetry which is clear in the form of (2.1)–(2.3).

In the numerical simulations that follow we set *b* = 10^−3^; this term is in fact not required but is helpful in avoiding computational issues and situations in which TF levels become unphysically negative. The parameter *α* controls the amount of twisting that trajectories undergo before they stabilize at one of three equilibrium states in which one of the TFs is large and the other two remain very close to zero. More precisely, the amount of twisting is a function of *α* − *β* but we fix *β* = 0.1 for simplicity and consider *α* in the range 10^−1^ ≤ *α* ≤ 10^3^. The reverse procedure, i.e. fixing *α* and varying *β*, would give equivalent, symmetrically related, results. Also, for simplicity, we fix *d* = 0.1 the rate at which the TFs degrade, and we set the exponent *h* in the Hill function to be *h* = 3, describing the nonlinear and saturating nature of the inhibitory response as the TF concentrations increase. Our results are not sensitive to these choices of *b*, *d* and *h*.

Finally, the function *g*(*t*) is taken to be time-dependent and describes exogeneous changes in the cellular environment, i.e. signalling activity, that drive the cell through the process of differentiation from an initial multi-potent state at time *t* = 0 into one of three differentiated states at large times (here taken to be *t* = 2000). We use the particular functional form

2.4 $$g(t)=\frac{4t}{t+2000},$$

and we consider the time interval 0 ≤ *t* ≤ 2000, so that *g*(*t*) varies from *g* = 0 at *t* = 0 to *g* = 2 when *t* = 2000.

**Figure 2.**
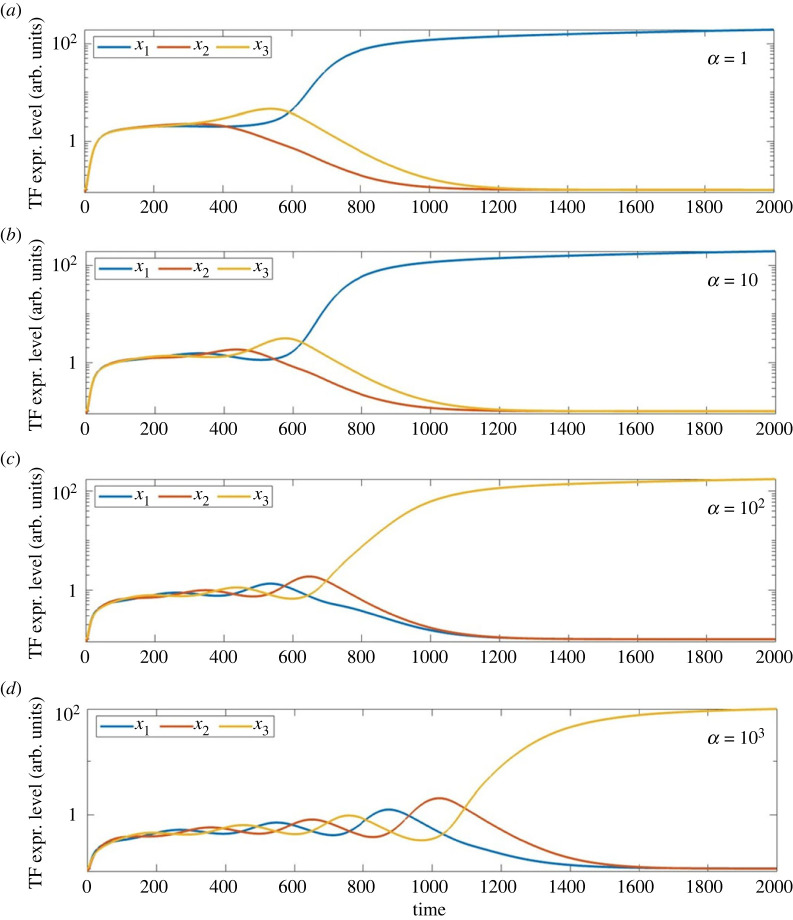
Typical trajectories of the deterministic (but non-autonomous) ODEs (2.1)–(2.3). Initially, all three variables *x*_*j*_(*t*) increase together but then separate in a manner that becomes increasingly oscillatory as *α* increases. In all cases, the trajectory tends to a state in which one TF dominates the other two. Parameter values: (*a*) *α* = 1; (*b*) *α* = 10; (*c*) *α* = 10^2^ and (*d*) *α* = 10^3^. All simulations use the same initial condition: (*x*_1_(0), *x*_2_(0), *x*_3_(0)) = (0.097, 0.09, 0.1).

The deterministic ODEs have well-understood behaviour that is illustrated in figure 2. For small fixed values of *g* (and hence also at small times in our non-autonomous simulations), there is a stable symmetric equilibrium point at which *x*_1_ = *x*_2_ = *x*_3_, i.e. the levels of the TFs are equal. As *t* (and therefore *g*) increases further, past approximately *t* = 400, this equilibrium point loses stability and trajectories move away from it in a spiralling motion that becomes increasingly apparent for larger fixed values of *α*. Eventually, one TF becomes larger than the other two, and then, via the inhibitory interactions shown in figure 1, suppresses them.

A linear stability analysis of the symmetric equilibrium point shows that the oscillations arise directly due to the Hopf bifurcation at which that equilibrium point loses stability. The imaginary parts of the pair of eigenvalues that cross the imaginary axis at the Hopf bifurcation are proportional to *α* − *β*, so for fixed *β* = 0.1, as we consider here, increasing *α* is expected to increase the oscillation frequency of trajectories as they leave the vicinity of the symmetric equilibrium.

To include stochastic aspects of both the intrinsic dynamics of a GRN and the nature of the observations that are typically obtained in scRNA-seq, we add a multiplicative stochastic term to the right-hand side of the ODEs (2.1)–(2.3). Note that the ODEs should be written as d*x*_*i*_ = *f*_*i*_(*x*_1_, *x*_2_, *x*_3_, *t*) d*t* for *i* = 1, 2, 3, where we include *t* explicitly as an argument in the function *f*_*i*_ since the ODEs are non-autonomous due to the term *g*(*t*) defined in (2.4). When we include the noise term, we have

2.5 $$dx_{i}=f_{i}(x_{1},x_{2},x_{3},t)\;dt+\sigma \sqrt{x_{i}}\;dW,$$

where *W* is a Wiener process with mean zero, unit variance and independent Gaussian increments. The multiplicative factor $\sqrt{x_{i}}$ in the noise term increases the absolute noise level as development proceeds, but produces greater variation at large TF expression levels than an additive noise term would, thereby amplifying the role of the noise compared with the purely additive case. We find that our results do not depend sensitively on the form of the noise term. To carry out the stochastic differential equation (SDE) integrations we use the implementation in Matlab of the Euler–Maruyama method written by Andrew D. Horchler and distributed as part of the SDETools package [28]. We use values for the noise level *σ* in the range from 0 to 0.02.

**Figure 3.**
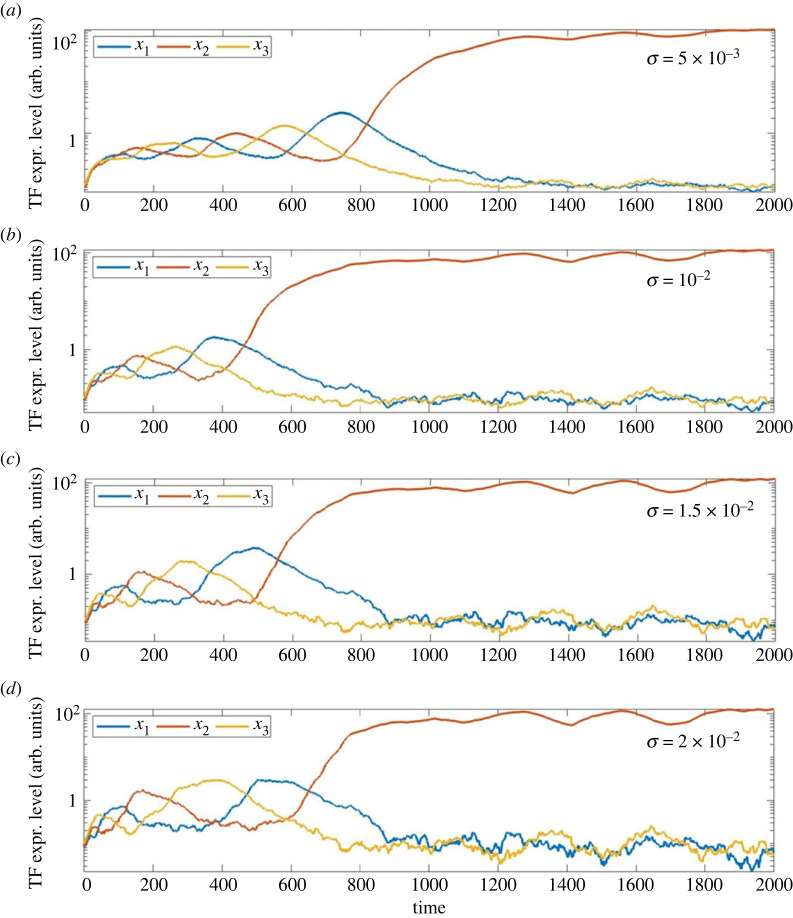
Typical sample paths of the stochastic ODEs (2.5) as *σ* increases for fixed *α* = 10^3^. (*a*) *σ* = 5 × 10^−3^; (*b*) *σ* = 10^−2^; (*c*) *σ* = 1.5 × 10^−2^ and (*d*) *σ* = 2 × 10^−2^. Compare with figure 2*d* in which *α* = 10^3^ also but where *σ* = 0.

Figure 3 summarizes the effect of increasing the noise amplitude *σ* while keeping *α* fixed. As one would expect the general pattern of the trajectories remains similar but the typical size of the stochastic fluctuations in the dynamics increases with *σ*. For this range of *σ*, the fluctuations preserve the fact that trajectories lie close to states where all three coordinates are equal at early times, and then tend to a state in which one TF ends up much larger than the other two, implying the selection of a particular cell fate, here identified exactly with one of the TFs taking a high value and the other two being expressed at much lower levels.

### 2.2. Synthetic data and *slingshot*

The *slingshot* R package is one of many that contains methods for inferring cell lineages and pseudotimes from single-cell gene expression data [13]. The data analysis workflow comprises three main steps:

1. First, we cluster the data. Technically this step does not require *slingshot*, and there are many possible clustering algorithms. As noted earlier we use the well-known *k-means* algorithm and discuss the effect of different clustering algorithms in §6.
2. Second, we generate lineages. As stated in [13], ‘Slingshot identifies lineages by treating clusters of cells as nodes in a graph and drawing a minimum spanning tree (MST) between the nodes ... Lineages are then defined as ordered sets of clusters created by tracing paths through the MST, starting from a given root node.’ Here, we explicitly identify the root node of the graph since we know from the dataset which points correspond to the earliest simulation times.
3. Third, for each path in the graph (i.e. each ordered set of clusters), *slingshot* generates a curve that passes through the clusters on each lineage, using a modified version of the principal curve algorithm [29].

In the numerical experiments we report on here, our interest lies primarily in the first two of these steps, and the question of whether *slingshot* is able to detect and reconstruct precisely three developmental lineages, where each lineage should connect early time multi-potent stem cells with one of the three terminal clusters that contains points with precisely one TF at a high level of expression.

## 3. Methodology

In this section, we discuss in more detail the data generation, clustering and lineage generation steps (steps 1 and 2 above). For most of the numerical experiments, we explicitly fix the starting cluster, since at early times, the levels of TFs along the trajectories increase monotonically and are subject to only small absolute levels of fluctuations due to the multiplicative nature of the noise term in (2.5).

### 3.1. Synthetic data generation

Fixing parameter values, we generate sample paths (trajectories) of the SDEs (2.5) for our synthetic dataset to input to the *slingshot* algorithm as follows. We require this data to look like a set of TF expression levels generated across the whole trajectory (for times 0 ≤ *t* ≤ 2000) and to represent equally (for simplicity) lineages that terminate at each of the three differentiated cell types, to mimic single-cell RNA-seq data from a large population of cells containing all three differentiated types. A single realization of the SDEs (2.5) corresponds in our conceptual model to the developmental journey of a single cell.

To ensure the generation of equal numbers of cells in the three lineages, we simply cyclically permute the coordinates of points on the trajectory. This generates in total a set of three trajectories (the original plus two further copies) from the original sample path. Regardless of which lineage the original trajectory followed, each of the three trajectories in the set must then terminate at a different one of the three distinct cell fates.

We then randomly sample 400 points from each of the three trajectories in order to build a set of synthetic cell states, uniformly distributed along each of the three differentiation pathways that our model describes. We export the time points and coordinates (*t*, *x*_1_, *x*_2_, *x*_3_) of these points to a .csv file that can be imported into *RStudio* to apply algorithms from the *slingshot* package. The synthetic input files therefore contain a set of 1200 points that we think of as representing the TF expression levels in a set of 1200 cells.

### 3.2. Clustering

For convenience we first rescale the data by working with the natural logarithms of the coordinates. This effectively enlarges the region around the multi-potent cell state and transforms trajectories into shapes that are easier to interpret.

We then apply *k*-means clustering function to the set of points (*x*_1*j*_, *x*_2*j*_, *x*_3*j*_), for *j* = 1, …, 1200 in $R^{3}$. *k*-means attempts to find an optimal set of clusters through an iterative process in which points are swapped from one cluster to another to minimize the ‘within-cluster-sum-of-squared-distances’ between the cluster centroids and the points in each cluster. We typically fix *k* to lie in the range from 5 to 40. With fewer than five clusters very few lineages can be constructed, since each must contain at least two clusters (the central one and a terminal cluster), while 40 is a sufficiently large number of clusters to illustrate the difficulties that are encountered in the large-*k* regime. Since distances are computed using the standard Euclidean metric, the resulting clusters tend to be roughly spherical neighbourhoods; *k*-means also favours clusterings in which the clusters are of roughly equal sizes. The results of a single run of *k*-means depend on the choice of initial starting choices for centroids, and potentially find only a local minimum and not the global minimum. So, as is standard practice, we run *k*-means multiple times (typically 50 instances, and up to 50 iterations in each run) and take the best clustering obtained. *k*-means does not determine an optimal number of clusters to use; we therefore use this as another input parameter and explore how the resulting typical number of lineages varies with the choice of *k*.

### 3.3. Computation of lineages

Having clustered the data, we apply the *slingshot* routines getLineages() and getCurves(). We identify explicitly the starting cluster for lineages: in experimental work. the starting cluster can often be deduced from physiological information; for our synthetic data, the identification of the earliest cluster is clear from the proximity of points to the initial condition (and the (1, 1, 1) axis), and this is consistent across our simulations.

**Figure 4.**
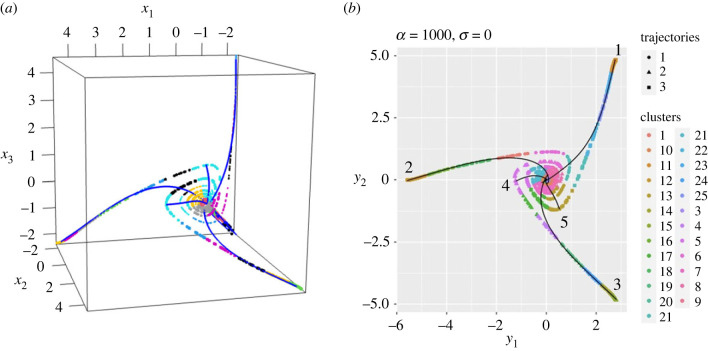
Typical result of a clustering and principal curves computation for synthetic data. Clusters are indicated by the colours of points, and the principal curves overlaid as solid lines (shown in blue in plot (*a*) and in black in plot (*b*)). In this case, the algorithm has detected five lineages, for clarity numbered in (*b*), starting from the origin, but only three extend out to the expected terminal clusters; the other two terminate close to the centre. (*a*) Data plotted in the original coordinates $(x_{1},x_{2},x_{3})\in R^{3}$, after taking logarithms; (*b*) data plotted in barycentric coordinates (*y*_1_, *y*_2_), i.e. in two dimensions. Parameter values: *α* = 1000, *σ* = 0, *k* = 25.

Figure 4 shows a typical output collection of clusters and lineages for a case in which there is substantial oscillation (*α* = 10^3^) but no multiplicative noise in the underlying differential equation (*σ* = 0). As an input we set the number of clusters *k* to be *k* = 25, but the number and form of the lineages is computed by the slingshot algorithm. We observe that all five lineages start in the lower-back-right of the three-dimensional plot figure 4*a* and begin to spiral outwards as they move upwards. Three complete lineages are identified by the algorithm, passing through clusters of points starting at the lower-back-right and terminating at approximately (4, −2, −2) and its permutations, in log-coordinates. These three are the lineages that we expected to recover. But in addition in this case there are two anomalous lineages that terminate closer to the origin and which we know, in this synthetic case, do not correspond to ‘true’ cell lineages. Figure 4*b* shows the same data plotted in two dimensions using the barycentric coordinates (*y*_1_, *y*_2_) defined by

$$y_{1}=\frac{x_{1}+x_{2}-2x_{3}}{\sqrt{6}}\;\text{and}\;y_{2}=\frac{x_{2}-x_{1}}{\sqrt{2}}.$$

The coordinates (*y*_1_, *y*_2_) are the natural coordinates to describe the data after orthogonal projection of points on to the two-dimensional plane *x*_1_ + *x*_2_ + *x*_3_ = const which clearly lies at right-angles to the vector (1, 1, 1)^*T*^. For this set of differential equations, this projection is natural since it is the one that maximizes the distance in the (*y*_1_, *y*_2_) plane between the three distinct differentiated cell states, analogous to the role that UMAP or principal component computations aim to do in general.

The use of the (*y*_1_, *y*_2_) coordinates brings out more clearly the dynamics of trajectories as they move away from the multi-potent stem cell state which now lies at the origin *y*_1_ = *y*_2_ = 0. The only drawback is the loss of information at early times when the three coordinates are nearly equal to each other but all three are increasing. For consistency, we therefore carry out the clustering and lineage calculations using the data points in the original (*x*_1_, *x*_2_, *x*_3_) coordinates even when we plot them using the barycentric coordinates (*y*_1_, *y*_2_).

**Figure 5.**
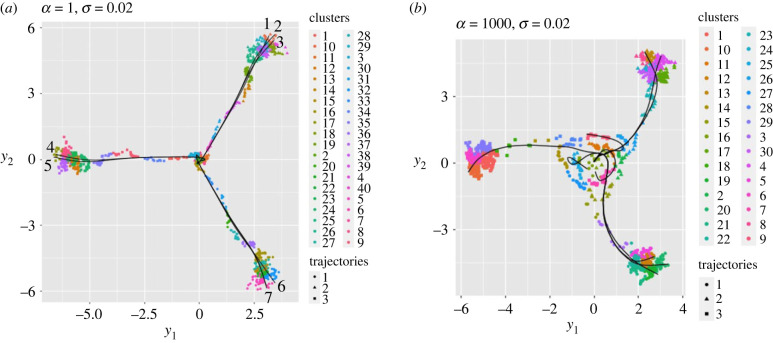
Illustrative results of clustering and principal curve computations using data generated in the presence of noise, plotted using the barycentric coordinates (*y*_1_, *y*_2_). (*a*) *α* = 1, corresponding to very little oscillation in trajectories, and *k* = 40 clusters. A total of seven lineages (indicated by the solid black lines which are their principal curves) are identified (and numbered): three extend to the top right corner, and two to each of the other terminal clusters on the left-hand side in the centre, and in the lower right-hand corner. (*b*) *α* = 10^3^, corresponding to a high degree of oscillation and *k* = 30 clusters. A total of eight lineages are identified: two terminate close to the centre, two at the upper right-hand corner, three at the lower right-hand corner and one at the left-hand centre terminal cluster. In both (*a*) and (*b*), the noise level *σ* = 0.02.

Figure 5 illustrates typical results obtained in the presence of noise, taking the parameter *σ* = 0.02. For the case *α* = 1 in figure 5*a*, we observe that seven lineages (indicated by the solid black lines which are their principle curves) are computed, rather than the expected three, but that all seven extend far from the origin and end at clusters that lie close to the expected fully differentiated states, identifying these with locations in which the expression level of one of the TFs is large and the levels of the other two are small. The existence of these seven lineages is due to the algorithm identifying more than three (indeed, seven) terminal clusters. In contrast, in figure 5*b*, there are eight principal curves but two of them terminate much closer to the origin than the other six.

### 3.4. Distinguishing two kinds of lineage

**Figure 6.**
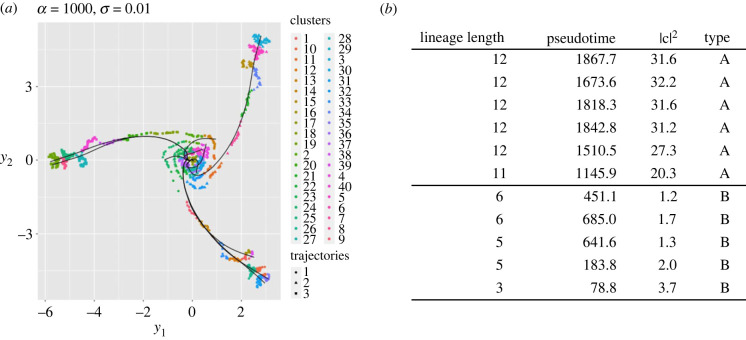
Typical example of type A and type B lineages, and summary statistics showing the clear distinction between the two types. Columns from left to right show: the length of the lineage, the elapsed pseudotime between the start and end clusters, the squared norm |**c**_*j*_|^2^ and the lineage type. Note that the first six lineages have significantly higher cluster length, time and squared norm. Parameter values: *α* = 10^3^, *σ* = 0.01 and *k* = 40.

We find that the above observation that there are two possible types of ‘anomalous’ lineage arises robustly. An illustrative example is given in figure 6 where we show one trajectory for the parameter values *α* = 10^3^, *σ* = 0.01 and *k* = 40 together with the clusters indicated by points of different colours. The computed values of |**c**_*j*_|^2^ for the clusters separate the lineages extremely well into six ‘long’ lineages that terminate far from the origin and five ‘short’ lineages that terminate much closer to it.

We refer to those lineages that have an end cluster that lies close to one of the true terminal states (near to one of the three axes) as type A, and those lineages whose end cluster lies much closer to the original multi-potent state as type B. Three distinct ways in which the data associated with type A and type B end clusters differ from each other are as follows:

(1) Type A end clusters contain points that have a higher mean (pseudo)time than those in a type B end cluster.
(2) Type A lineages are significantly longer paths in the underlying minimum spanning tree—i.e. they comprise more clusters connected in a chain—than those that have type B end clusters. The lineage length can be easily obtained from the output of the *slingshot* package.
(3) The centroids of end clusters for type A lineages have significantly larger squared Euclidean norm |**c**|^2^ than the centroids of type B clusters, reflecting the fact that type A lineages terminate much further away from the origin.

By squared Euclidean norm |**c**_*j*_|^2^ for cluster *j*, we mean the squared norm of the coordinate values of the centroid **c**_*j*_ = (*c*_1*j*_, *c*_2*j*_, *c*_3*j*_) after taking the logarithm. That is, we define

$$c_{ij}\;:=\sqrt{\frac{1}{N_{j}}\sum_{\ell \in I_{j}}\log_{e}x_{i\ell }},$$

where the sum is taken over points **x**_ℓ_ that lie in cluster *j*, as indicated by summing over the values of $\ell \in I_{j}$, where $I_{j}$ is the set of indices ℓ of points that lie in the *j*th cluster, and $N_{j}=|I_{j}|$ is the number of points in cluster *j* (i.e. the size of the index set $I_{j}$). We then define the squared Euclidean norm ${\left|c_{j}\right|}^{2}=\sum_{i=1}^{3}c_{ij}^{2}$.

Each of these three distinctions can be made quantitative by the imposition of an appropriate threshold above which we say the lineage is type A and below which it is type B (for pseudotime, lineage length and distance between the origin and the centroid of the terminal cluster, respectively). After detailed numerical experimentation we selected the third criterion as being the least affected by changes in the number of clusters *k*, the ODE oscillation parameter *α*, and the noise level *σ*. The lineage length criterion is clearly constrained by the choice of *k*, and we find in practice that the first criterion is more sensitive to the random generation of SDE sample paths, the random sampling of points and the precise values of *α* and *σ*. Hence, we define an end cluster, and therefore the lineage that it corresponds to, based on the third criterion:

— A lineage is defined to be *type A* if the centroid **c** of its terminal cluster satisfies ${\left|c\right|}^{2}>c_{0}^{2}$.
— A lineage is defined to be *type B* if the centroid **c** of its terminal cluster satisfies ${\left|c\right|}^{2}<c_{0}^{2}$.

Across all combinations of *α* and *σ* that we have surveyed, we find that for terminal clusters we rarely find values of |**c**|^2^ that lie in the range 4 to 16: the distinction between type A (for which usually |**c**|^2^ > 16) and type B (for which usually |**c**|^2^ < 4) is completely clear. We therefore set $c_{0}^{2}=8$ and conclude that our results are robust to this specific choice of threshold value.

## 4. Results

The distinction between type A and type B lineages made at the end of the previous section is important, because it transpires that the number of lineages of each type that is generated by the getlineage() routine behaves very differently as *α* and *σ* vary, as we now show.

**Figure 7.**
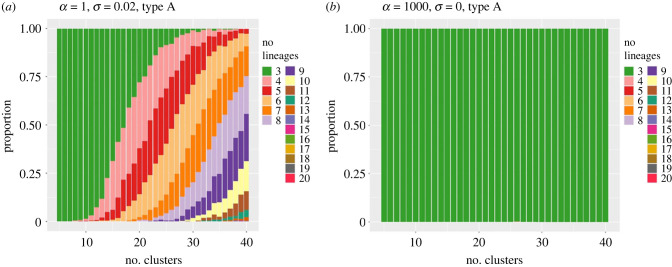
Stacked bar charts for the proportion of numbers of type A solutions detected in a set of 500 realizations of trajectories, as the number of clusters *k* increases. (*a*) case 1: *α* = 1, *σ* = 0.02; (*b*) case 2: *α* = 1000, *σ* = 0. In both plots, dark green indicates three lineages; pink = 4 lineages; red = 5 lineages, etc.

**Figure 8.**
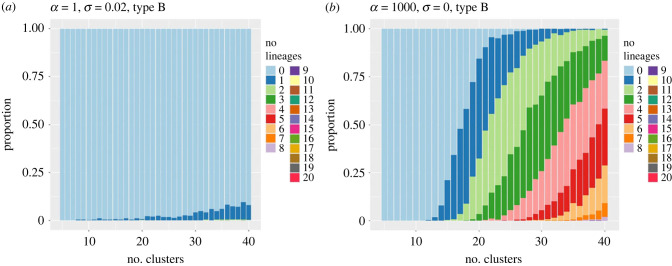
Stacked bar charts for the proportion of numbers of type B solutions detected in a set of 500 realizations of trajectories, as the number of clusters *k* increases. (*a*) case 1: *α* = 1, *σ* = 0.02; (*b*) case 2: *α* = 1000, *σ* = 0. In both plots, light blue=0 lineages, dark blue=1 lineage, light green = 2 lineages, etc.

In figures 7 and 8, we compare and contrast the results of a set of 500 realizations (for each combination of values of the parameters *α* and *σ*) of sampled data from SDE sample paths, with clusterings and lineage computations for each realization. The stacked bar charts show the proportion of those realizations in which a particular number of type A or type B lineages was detected as the number of clusters *k* was increased. Part (*a*) of each figure corresponds to the parameter values *α* = 1 (low oscillation) and *σ* = 0.02 (high noise level). As the number of clusters *k* increases, we see that the occurrence of additional type A lineages increases rapidly above three (the true number) as *k* rises above approximately 15 (figure 7*a*) yet the expected number of type B lineages remains very low (figure 8*a*).

In contrast, for parameter values *α* = 10^3^ (high oscillation) and *σ* = 0 (no noise), the number of type A lineages remains constant (at the expected value three) as *k* increases (figure 7*b*), while the number of type B lineages remains very low (zero or one) for *k* below 15, and then increases more rapidly with further increases in *k* (figure 8*b*).

Intuitively, in the low oscillation case, an increase in the number of clusters *k* introduces additional long (type A) lineages via ‘lineage splitting’ forming new but very similar lineages aligned with trajectories, but no new short type B lineages are generated. This allows the clustering to be adjusted by manually combining clusters (and indeed whole lineages with very similar endpoints) using additional expert judgement about whether differences between clusters are biologically meaningful or not.

In contrast, in the high oscillation, low noise case, there are consistently only three terminal clusters far from the origin (i.e. at most three lineages of type A can be constructed). From the requirement that the clusters are joined by a minimal spanning tree, additional clusters at other radial positions are then not able to ‘connect into’ the three type A lineages that persist as *k* increases, due to the requirement that the graph connecting clusters is a (minimal spanning) tree and so cannot contain cycles. Hence the number of type B lineages is almost forced to rise as *k* increases.

**Figure 9.**
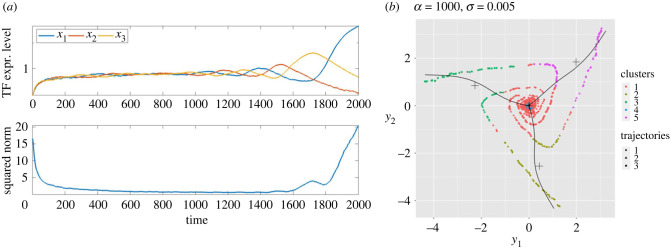
An atypical simulation run in which the trajectory spends a much longer time than usual spiralling, shown in (*a*), resulting in clusters that contain points from trajectories that have distinct terminal clusters, shown in (*b*). The three lineages are indicated by the three solid black lines which emerge from the centre and terminate towards the upper and lower right-hand corners, and more centrally on the left-hand side; these are the principal curves of these ‘lineages’. Parameter values: *α* = 1000, *σ* = 0.005.

We remark that the tendency of the *k*-means algorithm to create clusters that, even in the noise-free case, combine points from two or more trajectories as they spiral around is particularly well brought out in simulations containing long oscillatory transients, illustrated in figure 9. In such a case, the oscillatory nature of the intrinsic dynamics is clearly interfering with the ability of the *k*-means algorithm to construct a clustering that places points with the same ultimate differentiated fate into the same cluster.

**Figure 10.**
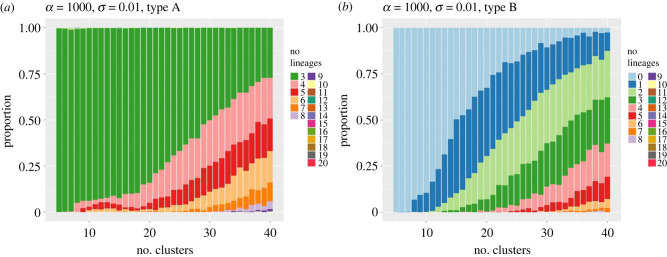
Stacked bar charts of the proportions of simulations in which different numbers of lineages were computed. (*a*) Type A lineages (dark green = 3 lineages, pink = 4 lineages, red = 5 lineages etc); (*b*) type B lineages (light blue=0 lineages, dark blue=1 lineage, light green=2 lineages etc). Parameter values: *α* = 1000, *σ* = 0.01.

In cases in which there is both significant oscillation (*α* = 10^3^) and significant noise (*σ* = 0.01), e.g. as shown in figure 10, we observe that the number of type B lineages rises much faster, as *k* increases, than the number of type A lineages. In other words, the presence of additional, anomalous, type B lineages is the dominant issue when a fine-grained clustering is performed. Naturally, in biological contexts there are often additional, for example physiological, markers of cell states which determine the relevant granularity of a clustering; these are absent here. As remarked on in the essay in [30], in some cases, there are indeed fundamental question as to whether a description in terms of discrete cell states makes sense, rather than a continuum through which the cell is continually moving.

**Figure 11.**
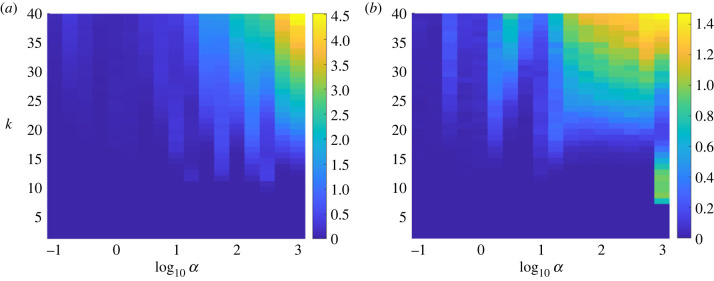
Heatmaps of the average number of type B lineages computed using the *slingshot* routines, for values of the ODE parameter *α* = 10^−1^, …, 10^3^ (logarithmic scale) and *k* = 1, …, 40 clusters. (*a*) *σ* = 10^−4^, averaged over 1000 realizations; (*b*) *σ* = 10^−2^, averaged over 500 realizations. Note that results in different columns are produced from independently sampled (randomly generated) data but results within a column are obtained from the analysis of the same collection of data and hence are highly correlated. The right-most column of (*b*) demonstrates that when the noise level *σ* is large, it is possible to generate, by chance, data that generate several type B lineages even when the clustering is coarse-grained, which then first disappear and then reappear as the number of clusters *k* is increased, even at large values of *α*.

As a result, we now focus on the formation of type B lineages, motivated by the formulation of a simple conceptual model that helps to explain the geometry of the construction of clusterings and lineages. This conceptual model can in fact be completely solved analytically and this forms the basis for the next section, where these analytical results explain the behaviour observed in figure 11.

## 5. Minimal geometrical models for trajectories

### 5.1. A specific example

In this section, we propose a very simple geometrical model which can be completely solved and sheds light on our numerical simulation results. In particular the model predicts that there exists a threshold below which, for sufficiently small levels of oscillation, i.e. |*α* − *β*| sufficiently small in the case of (2.1)–(2.3), the lineage calculations should return no anomalously short (type B) lineages, even if the number of clusters is large. Above this threshold, the number of clusters matters, and anomalous lineages will be generated for any fixed value of |*α* − *β*| above the threshold, if the number of desired clusters is sufficiently large. These conclusions are qualitatively independent of the noise level and the density of sampled points along the differentiation trajectories (i.e. the number of cells available in the scRNA-seq dataset).

A minimal conceptual model that captures the evolution of trajectories away from a multi-potent state towards one of several differentiated states is given by the differential equations

5.1 $$\frac{dr}{dt}=ar$$

and

5.2 $$\frac{d\theta }{dt}=\frac{b}{1+cr},$$

where *a*, *b* and *c* are positive constants, and (*r*, *θ*) are polar coordinates in the plane in which trajectories escape exponentially from the multi-potent state (which corresponds to the equilibrium point at *r* = 0). At long times, *θ* tends to a constant (since d*θ*/d*t* → 0 as *r* → ∞) which corresponds to motion that becomes radial far away from the origin. The parameter *b* controls how much spiralling occurs before this radial behaviour is reached (more precisely, the amount of spiralling is controlled by the ratio *a*/*b*). The parameter *c* controls the typical distance from the origin at which spiralling ceases.

Integrating (5.1)–(5.2) to compute the shape of trajectories *r*(*θ*), we find explicitly

5.3 $$r(\theta )=\frac{\exp\left(a\theta /b\right)}{c[1-\exp\left(a\theta /b\right)]},$$

where we fix a constant of integration by requiring that *θ* → 0 from below as *r* → ∞, i.e. selecting this trajectory out of a whole family of curves, all rotating in the positive (anticlockwise) sense since *b* > 0.

In order to avoid the generation of type B lineages, we must ensure that clusters are computed from points that lie close to each of three equally spaced trajectories that are described by (5.3) and its two rotationally symmetric images given by replacing *θ* in (5.3) by *θ* − 2*π*/3 or *θ* − 4*π*/3, respectively. For a given number of clusters *k*, as remarked on earlier, the *k*-means algorithm will produce clusters that are disc-like, and which have approximately equal radii, say *r*_0_. One cluster will be formed centred on the origin *r* = 0 and corresponding to multi-potent cells before differentiation has begun. The trajectory (5.3) passes through the point (*r*_0_, *θ*_0_) where

5.4 $$\theta_{0}=\frac{b}{a}\;\log\left(\frac{cr_{0}}{1+cr_{0}}\right),$$

which we note is negative, as we expect given that *θ* increases along the trajectory, and *θ* → 0 as *r* → ∞.

**Figure 12.**
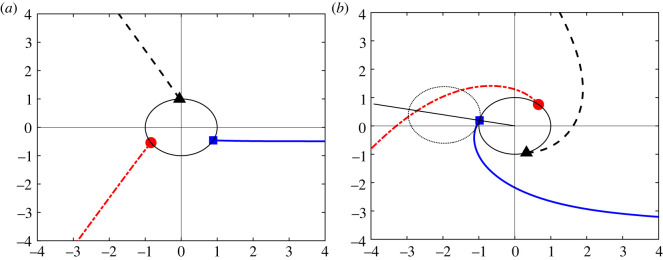
Illustrative trajectories that are asymptotic to straight lines extending from the origin at angles *θ* = 0, 2*π*/3, 4*π*/3 at large distances. The lines start at radius *r*_0_ = 1 and initial angles {*θ*_0_, *θ*_0_ + 2*π*/3, *θ*_0_ + 4*π*/3} (shown by the blue square, black triangle and red dot, respectively) where *θ*_0_ is defined by (5.4). (*a*) The case *b* = 0.5 corresponding to a small amount of oscillation; (*b*) The case *b* = 3.5 for which there is much greater twisting of trajectories. In both cases, we set *a* = 0.1 and *c* = 10.

Figure 12 shows two sets of trajectories that are defined by (5.3) and start at *r* = *r*_0_ and *θ* ∈ {*θ*_0_, *θ*_0_ + 2*π*/3, *θ*_0_ + 4*π*/3}, where *θ*_0_ is determined by (5.4). The point (*r*_0_, *θ*_0_) is indicated by the blue square. As *b* increases at fixed *a*, it is clear that the red dot-dashed trajectory that starts from the red circle placed at (*r*_0_, *θ*_0_ + 4*π*/3) passes closer to the blue square (and similarly for the other trajectories as they are symmetrically related), indicating that there is a greater likelihood that points lying close to two different trajectories will be wrongly clustered together, or that the clustering algorithm will introduce new erroneous (type B) end clusters that will not correspond to a full cell lineage.

Therefore, to ensure that only three lineages are formed moving away from the origin, we require that the trajectory starting at (*r*_0_, *θ*_0_) is well-separated from the similar trajectory that starts at (*r*_0_, *θ*_0_ + 2*π*/3) which tends towards the *next* of the three terminal differentiated states, placed at *θ* = 2*π*/3, as *r* → ∞. Well-separated here could be interpreted as the condition that the trajectory (5.3) lies at all times at least one cluster diameter, i.e. a distance 2*r*_0_, away from its symmetrically related counterparts. These distances are smallest at small distances to the origin, so the condition for these three trajectories to all be well-separated from each other becomes

5.5 $$r(\theta_{0}+2\pi /3)-r_{0}\geq 2r_{0}.$$

This constraint is indicated in figure 12*b* where the red dot-dashed line cuts the ray from the origin at angle *θ*_0_ (i.e. the straight line that passes through the blue square at (*r*_0_, *θ*_0_)) inside the dotted circle (which has diameter 2*r*_0_) rather than cutting it outside the circle as would be case for smaller *b*. The constraint for well-separated trajectories, (5.5), becomes

5.6 $$\frac{e^{(\theta_{0}+2\pi /3)a/b}}{c(1-e^{(\theta_{0}+2\pi /3)a/b})}\geq 3r_{0}.$$

Rearranging this inequality and using (5.4), we obtain

5.7 $$r_{0}\geq \frac{3-E}{3c(E-1)}=:\;R(E),$$

where E=exp⁡(2πa/(3b)) is a measure of the ratio of the rate of radial expansion of trajectories to their rotation rate around the origin, and (for later convenience) we define the right-hand side of the inequality to be the function *R*(*E*). Since *E* > 1 always, we conclude that the constraint (5.7) describes two slightly different sub-cases.

For the first case consider *E* > 3; then the right-hand side of (5.7) is negative and so the inequality holds for all values of *r*_0_, i.e. all cluster sizes, equivalently all numbers of clusters *k* since the size of a typical cluster will decrease in proportion to the number of clusters: points are distributed mostly along linear segments of constant length and so *r*_0_ ∝ 1/*k*.

In the second case, 1 < *E* < 3, the trajectories are well-separated only if *r*_0_ is large enough (i.e. the number of clusters *k* is small enough). The critical case *E* = 3 corresponds to a≥(3log⁡3)/(2πb) which is independent of the parameter *c* and rather conveniently, given that the constant (3log⁡3)/(2π)≈0.52, suggests that the number of clusters starts to matter when the rate *b* of azimuthal motion around the origin becomes as large as (roughly twice) the radial rate of separation *a*.

**Figure 13.**
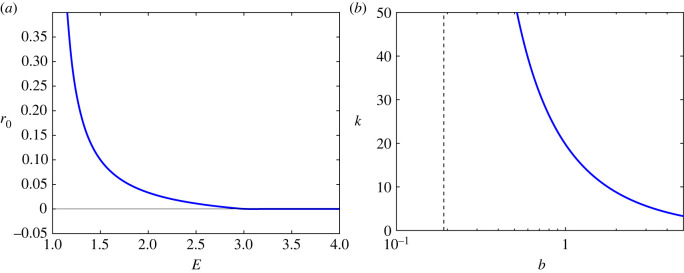
Illustration of the regimes in which (5.5) is satisfied; for sufficiently large *r*_0_ in (*a*) and sufficiently small *b* in (*b*). (*a*) Trajectories are well-separated above the blue curve which takes the form $r_{0}(E)=\max(R(E),0)$ with *R*(*E*) as defined in (5.5). (*b*) Trajectories are well-separated below the blue curve which has an asymptote at b=2πa/(3log⁡3) indicated by the vertical dashed line. For comparison with figure 11. Parameter values: *a* = 0.1 and *c* = 10.

Figure 13 plots the boundary of the regime in which trajectories are well-separated, in both the (*E*, *r*_0_) plane and the (*b*, *k*) plane. We observe that as the rate of rotation *b* around the origin decreases, the maximum number of clusters for which trajectories do not interfere with each other increases, and for values of *b* below the critical value 2πa/(3log⁡3) there is no maximum number of clusters. This qualitatively agrees with figure 11, which indicates that the number of type B lineages (indicative of trajectories not being well-separated) is very small, for any number of clusters *k*, below a threshold value of *α*. The parameters *α* and *b* play the same role in controlling the amount of rotation of trajectories in the original model (2.1)–(2.3) and in the conceptual model (5.1)–(5.2), respectively, so this agreement is extremely encouraging.

### 5.2. A general theorem

We now consider replacing the specific model (5.1)–(5.2) with differential equations of the form

5.8 $$\dot{r}=af(r)\;\text{and}\;\dot{\theta }=bg(r),$$

where the constants *a* and *b* are positive and *f*(*r*) and *g*(*r*) are assumed to be continuously differentiable for 0 ≤ *r* < ∞, and to satisfy the following conditions:

— *f*(0) = 0 and *f*′(*r*) ≥ 0,
— *g*(0) = 1, *g*(*r*) > 0, *g*′(*r*) ≤ 0 and *g*(*r*) → 0 as *r* → ∞.

We define the function *F*(*r*) for 0 ≤ *r* < ∞ as

5.9 $$F(r)\;:=\exp\left(-\int_{r}^{\infty }\frac{g(s)}{f\;(s)}\;ds\right).$$

Then we have the following result.

**Theorem 5.1.**

*Let our model for trajectories be defined by* (*5.8*) *for choices of* *f* *and* *g* *that satisfy the conditions given above, and with* *F*(*r*) *as defined in* (*5.9*). *Then if* *F* *is concave, trajectories are always well-separated when*

5.10 $$a\geq \frac{3\log3}{2\pi }b.$$

It is notable that this theorem revolves around the same inequality as we computed directly for the specific choice of *f* and *g* used in the previous subsection, even though *f* and *g* are not prescribed (apart from satisfying the conditions given above). This adds support to our view of the generic, and geometric, nature of the previous result.

**Proof.**

First, we note that the conditions on *f* and *g* specified above imply that the origin is an equilibrium point and that *g*(*s*)/*f*(*s*) is positive, monotonically decreasing (since (d/d*s*)(*g*(*s*)/*f*(*s*)) ≤ 0 for *s* > 0), and

$$\frac{g(s)}{f\;(s)}\to 0\;\text{as}\;s\to \infty \;\text{and}\;\frac{g(s)}{f\;(s)}\to \infty \;\text{as}\;s\to 0.$$

Then, writing

$$\frac{dr}{d\theta }=\frac{\dot{r}}{\dot{\theta }}=\frac{af\;(r)}{bg(r)}\;$$

and integrating we obtain

5.11 $$\int_{\infty }^{r}\frac{g(s)}{f\;(s)}\;ds=\frac{a}{b}\theta ,$$

where we have imposed the boundary condition that *θ* → 0 as *r* → ∞. We write this as

$$F(r)\;:=\exp\left(-\int_{r}^{\infty }\frac{g(s)}{f\;(s)}\;ds\right)=\exp\left(\frac{a\theta }{b}\right)\;$$

and note that *F*(0) = 0, *F*(*r*) is monotonically increasing, and *F*(*r*) → 1 as *r* → ∞. Therefore, *F*^−1^ exists and it makes sense to write

$$r=F^{-1}\left(\exp\left(\frac{a\theta }{b}\right)\right),$$

which is the generalization of (5.3).

Now we apply the same geometric condition for trajectories to be well-separated, i.e. (5.5). In this more general context, we still consider a trajectory that starts at the initial point (*r*_0_, *θ*_0_) where $\exp(a\theta_{0}/b)=F(r_{0})$. Recall that *r*_0_ is the radius of a typical cluster, and hence the radius of the cluster containing points near origin, corresponding to the multi-potent cell state. The constraint (5.5) now becomes

$$r(\theta_{0}+2\pi /3)=F^{-1}(e^{(\theta_{0}+2\pi /3)a/b})\geq 3r_{0},$$

which, since *F*^−1^ exists, is equivalent to the condition that

5.12 $$F(r_{0})\;e^{2\pi a/(3b)}\geq F(3r_{0}),\;\text{i.e. that}\;\frac{F(3r_{0})}{F(r_{0})}\leq e^{2\pi a/(3b)}.$$

Now we recall that *F*(*r*) being *concave* for 0 ≤ *r* < ∞ means that for all *x*, *y* > 0 and 0 ≤ *λ* ≤ 1,

λF(x)+(1−λ)F(y)≤F(λx+(1−λ)y).

Geometrically, this means that straight lines joining points on *F* always lie below the graph of *F*.

Setting *y* = 0, *x* = 3*r*_0_ and *λ* = 1/3 this implies that

5.13 $$\frac{1}{3}F(3r_{0})\leq F(r_{0})\;\text{and hence}\;\frac{F(3r_{0})}{F(r_{0})}\leq 3,$$

for all *r*_0_. Combining (5.12) and (5.13), we see that if 3 ≤ e^2*πa*/(3*b*)^ then the statement that *F* is concave implies that (5.12) holds and therefore that trajectories are well-separated. Hence, trajectories are well-separated for all *r*_0_ (i.e. for any number *k* of clusters) when (5.10) holds. ▪

**Remark 5.2.**

We remark that a sufficient condition for *F* to be concave would be that

5.14 $$F^{\prime \prime }(r)=F(r)\left[{\left(\frac{g(r)}{\;f\;(r)}\right)}^{2}+\frac{d}{dr}\left(\frac{g(r)}{\;f\;(r)}\right)\right]\leq 0,$$

but that from the assumptions on *f* and *g* above the two terms in the square brackets will in general have opposite signs and so we cannot directly conclude that *F* will always be concave.

For the specific model (5.1)–(5.2), a direct calculation shows that

$$F^{\prime \prime }(r)=\frac{-2rF(r)}{{\left[r(1+r)\right]}^{2}}\leq 0,$$

and so in this case *F* is indeed concave and so the theorem applies, as we have seen by direct calculation.

**Remark 5.3.**

If we change the set-up of the problem so that there are *N* ≥ 2 fates, rather than three, and we assume that there are therefore *N* symmetrically placed terminal clusters instead of the three assumed in the conceptual model outlined in the paper, then, under the assumptions above, we would expect the result (5.10) to be replaced by

5.15 $$a\geq \frac{N\log N}{2\pi }b.$$

This is a straightforward generalization of the proof above, replacing 3 by *N* throughout.

In summary, the above analysis serves to strengthen further our argument that short lineages are expected to arise due to a sufficiently high degree of twisting in the trajectories followed by differentiating cells, if the cells are clustered into a large number of clusters (i.e. *k* is taken to be large so that *r*_0_ is small).

## 6. Discussion and conclusion

In this paper, we have considered the effect of intrinsic oscillations in models of the dynamics of TFs governing cell fate choice. We have shown that even low levels of oscillatory behaviour are extremely likely to cause lineage reconstruction algorithms to generate spurious results if the data is subjected to a fine-scale clustering. As a result the data can only be clustered in a coarser way which naturally obscures the oscillatory dynamics that are driving the dynamics underneath. And the oscillatory dynamics leads to a patch of indeterminate data near the bifurcating branches of fate-restricted lineages in ways that are not simply due to noise or measurement error.

We generated synthetic data from our simple, and novel, conceptual model for cell fate differentiation that was proposed and analysed in our earlier work [16,20,21] in connection with a possible resolution of the long debate in the literature over the details of cell fate specification in the neural crest. The two schools of thought concern whether or not there exist partially restricted intermediate cell states between fully multi-potent and fully fate-committed states. In the direct fate restriction (DFR) paradigm there are no such intermediate states, while in the progressive fate restriction (PFR) paradigm they do exist. Our modelling and experimental work suggests that there is a third possibility which we have termed cyclical fate restriction (CFR). The model does not reproduce all the known features of data from genetic regulatory networks, but is simple enough to analyse in substantial detail and highlights the underlying role that the geometry of trajectories, rather than noise, plays. The model that we use is not at all intended to capture all relevant details of the biology; it is mathematically much simpler, and formulated so that we understand and can fully anticipate its expected behaviour. Of the very many elements that could be improved, one obvious one (which is likely to lead to only a very marginal improvement in practice, we believe) would be to replace our methodology to ensure equal numbers of points sampled from trajectories that are heading to each of the three cell fates by generating three copies of each of 400 simulations and then sampling at independent times from each, by sampling from a set of 1200 trajectories generated fully independently (but checking that out of the whole set, 400 trajectories terminated at each of the three cell fates).

It is interesting to note that a phenomenologically similar debate can be seen in relation to haematopoietic stem and progenitor cells (HSPCs) in mouse and human, as recently summarized in [31]. In particular, the results of [32] suggest that there is a ‘continuum of low-primed undifferentiated haematopoietic stem and progenitor cells’ which they refer to as ‘CLOUD-HSPCs’ from which fate-restricted cells emerge directly without passing through intermediate partially fate-restricted cell types. Further, the authors of [33] include the following in the abstract of their paper: ‘We show that each cell explores (at its own pace and independently of cell division) many different possibilities before reaching a stable combination of genes to be expressed.’

These papers therefore appear to describe situations which mirror observations in the literature on zebrafish neural crest, which provided the motivation for our modelling here. Moreover, the results of [34] indicate a possible shift in the mechanism by which cell fates are determined, again in the context of haematopoiesis, from a more clearly branched ‘PFR-like’ fate restriction process in fetal differentiation, towards a more ‘DFR-like’ process in adult human haematopoietic stem cells. More generally, the dynamic nature of gene expression levels in stem cells has been widely observed and remarked on, for example in mouse embryonic stem cells in [35].

We have explored how the oscillatory dynamics present in a model for CFR lead to complications in the standard data analysis pipeline for single-cell RNA sequence data and the reconstruction of cell fate lineages using the data processing routines in the standard bioinformatics package *slingshot*. The huge advantage of working with synthetic data from our model differential equations, of course, is that we know the true answers and so can compare the performance of these algorithms in a highly controlled situation. The drawback with synthetic data is that it does not capture the statistical behaviours present in real scRNA-seq data. It would be of substantial interest in future work to repeat the kind of analysis presented here using real biological data, or with synthetic data from a package such as Splatter [36] which can generate simulation data better able to fit observed distributions. It is worth noting, however, that in order to use (e.g.) the Splat simulation package from Splatter to generate synthetic data for the lineage reconstruction problem that we consider here, we would need to make a large number of choices about the input parametrizations of the differential expression levels for the genes, effectively coding in the mean trajectories such as those shown in figure 2, as well as measures defining the variance and ‘skewness’ (here meaning whether data points are more likely to come from one end or the other end of the lineage path) in order to drive the simulation. Without much more detailed guidance from a real experimental dataset, this process would feel rather subjective and perhaps difficult to justify in detail; resulting in the end in not much additional enlightenment compared with the mathematically simple and conceptual approach that we pursue here.

Within a high-dimensional genetic regulatory network (i.e. before dimension reduction techniques have been applied) there may well be additional effects, such as variations in the degradation rates for different genes controlled by the same TF, that would provide additional information on the relative timings of different cell states. In some sense, we collect all this environmental information, and information that might be present outside the ‘core GRN’ and represent it by the single function *g*(*t*). It is clearly of interest for future work to improve on that simple characterization.

Similar points of simplicity and mathematical clarity lie behind our choice of *k*-means as a clustering algorithm: it is well-known and, since the number of clusters can be specified in advance, we take advantage of that to explore the dependence of the results on the number of clusters. However, a popular approach in practice is to combine the shared-nearest-neighbour (SNN) algorithm to produce an initial clustering that is then refined with the Louvain algorithm. The SNN algorithm was proposed in [37] and later refined by other authors (e.g. [38,39]). While *k*-means tends by construction to produce circularly symmetric clusters (in two dimensions; spherically symmetric ones in higher dimensions), the SNN algorithm computes similarities between points based on the number of nearest neighbours that are shared by the two points. This feature naturally allows for a clustering of non-uniform distributions of points into clusters of different sizes. The ‘Louvain’ algorithm [40] is the process of testing the optimality of a given clustering by moving single points between clusters if such moves give a better clustering, as measured by the modularity of the clustering. Together this approach tends to produce clusters that are better able to adapt to the shape of the data which one might imagine in cases such as these follow more linear, elongated, shapes. This intuition is correct, however, in practice, data are never noise-free and all clusterings will follow similar geometric rules. The behaviour of the clustering algorithm is simpler to explore when the *k*-means algorithm is used, especially since the essence of our argument is indeed geometrical rather than linked to any particular clustering algorithm.

The standard paradigm for cell differentiation is that there is an almost-monotonic departure from the multi-potent state towards differentiated states, corresponding to the situation shown in figure 5*a*. The effect of increasing the number of clusters in *slingshot* in this case, where there is no twisting of trajectories, is typically to generate a small number of additional type A lineages, where the end clusters are distinct (as shown in figure 5*a*) but typically (i.e. over approximately 90% of the time) not to generate any type B lineages, even for (perhaps excessively) large numbers of clusters (e.g. up to *k* = 50 clusters), as shown in figure 5*b*. In real analyses, careful consideration of which TFs are expressed in different end clusters would be used to resolve the issue of multiple very similar-looking lineages and produce biologically reasonable results. Since very few type B lineages would be generated, the issue of their resolution does not arise.

However, if the expression-level data are generated through an oscillatory process then the issue of spurious type B lineages becomes much more important. Consider, for example, figure 10. In part (*a*) of this figure, we see that, up to approximately *k* = 17 clusters, approximately 90% of the time *slingshot* will identify (correctly) three type A lineages. But it will also generate one or more type B lineages, which we know in this synthetic example are spurious. Most ambitiously, it might be possible to use the variations in how the numbers of type A and type B clusters vary with *k* as a way of detecting the presence of oscillatory dynamics in real data.

A central aspect of the interest in this conceptual model is that it is often difficult, due to the twisting of trajectories, to give a good definition of the time at which the ‘decision is taken’ to commit to one or other cell fate. Mathematically speaking, the time at which the cell fate decision is made is determined through a combination of its initial condition, the value of the parameter *α* which controls the amount of twisting of trajectories, and the parameter *σ* which controls the level of noise along the trajectory. Figure 2 indicates that as *α* increases the ‘decision time’ shifts from approximately *t* = 600 to more like *t* = 1000, since as *α* increases the trajectory spends longer in its oscillatory phase before the expression level of a single TF becomes dominant. However, figure 3 suggests that with increasing levels *σ* of stochastic noise, the dominant TF (and hence the fate commitment) becomes clearer at slightly earlier times. And there are from time to time outliers as illustrated by figure 9.

In the experimental literature, we note that there are examples [14,41] of scRNA-seq data for cell differentiation in which a relatively large and amorphous central group of cells appears. For example, these are referred to as a ‘Hub’ cluster in [14]. In light of the issue above, we propose that these might be an example in which the cells are undergoing some kind of oscillatory or cyclical exploration of possible fates to commit to, and that this temporal oscillation causes the clustering and lineage computations difficulties in precisely the ways we have outlined above.

More generally, we have provided an example in which we can demonstrate that the presence of intrinsic oscillations in gene expression dynamics adds significant complexity to the task of interpreting single-cell RNA-seq data and the reconstruction of cell lineages; these issues are highly likely to recur in many situations and serve as motivation for the continued development of bioinformatics methods for pseudotime-based trajectory reconstruction.

One possible direction to help resolve this dynamical complexity would be to use data collected from more than one point in real time to help with the pseudotime reconstruction. The Waddington-OT method introduced in [42] both points towards a way of doing this, at least for cases in which gene expression levels diverge monotonically as different fates become specified, while also highlighting explicitly the inadequacies of pseudotime reconstruction methods based only on data at a single time point (as noted in §1 above). For biological processes that are well-known to oscillate, not least the cell cycle, progress can be made by building statistical models, such as the Oscope approach [43], that explicitly look for the presence of correlated oscillations between gene expression levels. The identification of pairs of genes that oscillate at the same frequency, but with a possible phase difference between them, can be detected if sufficiently large datasets are available and if there is sufficient regularity in the dynamics—for example the oscillations in expression levels have a constant amplitude. The direct application of the Oscope approach to our model is made extremely difficult by the transient nature of our oscillations; Oscope as currently developed is not able to distinguish between different cycles of an oscillation since it effectively looks to integrate information from across whole periods of oscillation.

However, it might be possible to combine elements of these different approaches, for example looking for oscillatory relationships between expression levels for pairs of TFs where information from multiple real-time points was available, in order to be able to assess the start and end of an oscillatory regime in the dynamics. There would seem to be significant value in using synthetic data initially to test the development of methods that explicitly take temporal information into account, in order to understand their potential scope, and protocols to tune their parameters, and through that to learn more fully their limitations when applied to real data, and criteria for the possible success of more complex lineage reconstruction methods.

## Data Availability

Code supporting this article is available as electronic supplementary material and also from the Zenodo digital repository: https://doi.org/10.5281/zenodo.10673541 [44]. Supplementary material is available online [45].
